# A possible mechanism for PTSD symptoms in patients with traumatic brain injury: central autonomic network disruption

**DOI:** 10.3389/fneng.2013.00013

**Published:** 2013-12-19

**Authors:** John B. Williamson, Kenneth M. Heilman, Eric C. Porges, Damon G. Lamb, Stephen W. Porges

**Affiliations:** ^1^Brain Rehabilitation Research Center, Malcom Randall VA Medical Center, McKnight Brain Institute, University of FloridaGainesville, FL, USA; ^2^Institute of Aging, Cognitive Aging and Memory Clinical Translational Research Program, University of FloridaGainesville, FL, USA; ^3^Department of Neurology, University of FloridaGainesville, FL, USA; ^4^Department of Psychiatry, University of North Carolina at Chapel HillChapel Hill, NC, USA

**Keywords:** emotion, autonomic nervous system, traumatic brain injury, post traumatic stress disorder, white matter, TBI, PTSD

## Abstract

Patients with traumatic brain injuries (TBI) often develop post traumatic stress disorder (PTSD). This syndrome, defined and diagnosed by psychological and behavioral features, is associated with symptoms such as anxiety and anger with an increase of arousal and vigilance, as well as flashbacks and nightmares. Many of these features and symptoms observed in PTSD may be in part the result of altered autonomic nervous system (ANS) activity in response to psychological and physical challenges. Brain imaging has documented that TBI often induces white matter damage to pathways associated with the anterior limb of the internal capsule and uncinate fasciculus. Since these white matter structures link neocortical networks with subcortical and limbic structures that regulate autonomic control centers, injury to these pathways may induce a loss of inhibitory control of the ANS. In this review, the autonomic features associated with PTSD are discussed in the context of traumatic brain injury. We posit that TBI induced damage to networks that regulate the ANS increase vulnerability to PTSD. The means by which the vulnerability can be measured and tested are also discussed.

## INTRODUCTION

The prevalence of mild traumatic brain injury (mTBI) and subsequent development of emotional disorders, most notably post traumatic stress disorder (PTSD), is high in veteran populations. We propose a mTBI-induced disruption of neural systems underlies this cluster of emotional disorders as well as the emotional and behavioral commonalities between PTSD and mTBI. The causes and mechanisms underlying this painful and disabling disorder are not sufficiently understood. Does mTBI contribute to the symptom complex that constitutes PTSD? Is this contribution due to direct neuronal injury (e.g., axonal shearing)? Do the experiences surrounding the occurrence of mTBI (e.g., combat roles resulting in injuries), influence the presentation of this syndrome? Behavioral neuroscience methodologies and recent advances in structural and functional imaging, provide a toolkit to identify and understand the neuroanatomical structures that are vulnerable to mTBI. Damage to certain commonly affected structures after mTBI may compromise regulation of autonomic networks that influence emotional state and directly influence the development of PTSD.

Below, the nature of mTBI is reviewed, including injury characteristics, associated measurable physiological changes, and epidemiological data on the impact of mTBI on behavior. We discuss emotion within the context of autonomic nervous system (ANS) driven defense and socially adaptive physiological states. PTSD is conceptualized as a core deficit in physiological state (i.e., a chronic defensive state manifested by hypervigilance and increased sympathetic drive and parasympathetic withdrawal). We develop the postulate that this aberrant physiological state induced by mTBI injury to white matter structures involved in limbic/ANS networks enhanced the vulnerability for development of PTSD.

**FIGURE 1:**
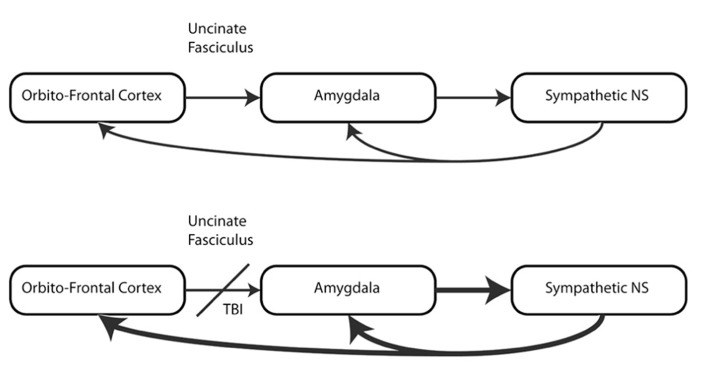
**Simplified network disruption example in mild TBI with symptoms of PTSD**.

The objective of this review is to examine the hypothesis that mTBI, through damage to white matter structures that control autonomic regulation, creates a vulnerability to the development of symptoms of PTSD, and to describe the technical advances that allow us to test this hypothesis (see **Figure 1**).

## MILD TRAUMATIC BRAIN INJURY

Head injury, and in particular mTBI, is common in both civilian and military populations. However, the extent and predictors of disability caused by mTBI are not fully known. A study of data from the 1991 National Health Interview survey estimated that about 25% of individuals with a self-reported traumatic brain injuries (TBI) that resulted in loss of consciousness did not seek medical attention (Sosin et al., 1996). In a more recent review of the incidence and presentation of TBI in the United States (Summers et al., 2009), it was noted that a diverse representation of age-groups and etiologies exists, including falls in the elderly, the most commonly injured group in the civilian population in this study, and blast injuries in the military population (97% of injuries in returning marines were from improvised explosive devices or mines). Other civilian studies suggest young adults (ages 18–25) are most likely to incur an mTBI from motor vehicle accidents and sports-related activities (Alexander et al., 2007). Hoge et al. (2008) reported a survey of two US Army infantry brigades (2,714 soldiers surveyed 3–4 months after conclusion of a 1 year deployment in Iraq) and found that roughly 15% of the troops reported an injury resulting in loss of consciousness or altered mental status. In another survey of 1965 service members, 19.5% reported a probable mTBI. The consequences of these mTBIs may be profound. Notably, of those 19.5% who reported TBI, 37.4% developed PTSD or depression (Tanielian and Jaycox, 2008). The deficits in behavior (cognitive and emotional) as a consequence of mTBI or multiple mTBIs have not been entirely elucidated and there remains conflicting evidence and confounding issues, such as secondary gain, premorbid medical and psychological disorders, and comorbid vulnerabilities (e.g., Ruff, 2009). These complications in evaluating the population necessitate a multi-layered approach including understanding of neurophysiological effects of TBI and how they may be related to PTSD.

## PATHOPHYSIOLOGY OF MILD TRAUMATIC BRAIN INJURY

Although mTBI may contribute to severe psychological disorders, these injuries and their outcomes are highly heterogeneous. Mild TBI may induce a transient loss of consciousness (<20 min) with anterograde or retrograde amnesia, with apparent recovery of cognitive functioning within 24 h. By definition, there are no focal neurological deficits. Usually, there are also no visible changes on conventional structural magnetic resonance imagine (MRI) studies, e.g., T1, T2, and FLAIR protocols. Current trends in research on the pathophysiology of mTBI appear to be leaning heavily to changes in white matter integrity, and cutting edge techniques for measuring white matter has allowed for more sensitive measurement of white matter injury. One mechanism of white matter injury in mTBI is diffuse axonal injury (DAI). The nature and location of DAI may be variable and, as such, may produce different, even conflicting, clinical outcomes. Further, the mechanism of injury may influence the anatomic distribution of white matter injuries and these differences in distribution may result in different vulnerabilities (e.g., blast-related TBI versus impact injury).

Diffuse axonal injury occurs due to angular and/or rotational acceleration that overwhelms the protective mechanisms of the skull, cerebrospinal fluid, and meninges. This acceleration is thought to result in shear and tensile strains in axons, resulting in damage to the architecture of white matter. Further, rapid acceleration, as in blast injury induced mTBI, may produce transient mechanical disruption of axonal membranes, leading to a cascade of chemical processes that disrupt the axonal cyto-architecture. While the location and severity of white matter damage may vary depending on the vector of force of the acceleration and impact (if applicable), there are regions that are particularly vulnerable and commonly affected after TBI, including the corpus callosum and subcortical white matter. In general, the longer and larger a white matter tract, the more vulnerable it will be to these effects of TBI. Relevant to PTSD and emotional disorders in general, fronto-subcortical networks are quite vulnerable to the effects of mTBI and even if they are spared direct injury, as secondary connections made through other white matter tracts may be damaged, the end result could still be compromised frontal lobe control even if direct connections are spared.

White matter damage after TBI is heterogeneous and this heterogeneity may partially explain differences in outcome for patients with mTBI. However, even though the injury is heterogeneous, certain brain regions appear to be more vulnerable than others and this vulnerability may place those that have sustained mTBI at greater risk for the development of emotional disorders. For example, Niogi et al. (2008), examined the extent and specificity of microstructural white matter injury in mTBI, comparing 34 patients with mTBI to 26 healthy controls and defining damage in any specific structure as DTI-FA values 2.5 SDs below this region’s average in normal subjects. They found that 41% of patients had damage to the anterior corona radiata and 30% had damage to the uncinate fasciculus. These white matter tracts appeared to be the most frequently damaged white matter structures in this population. Behaviorally, these changes in white matter were significantly related to performance on reaction time tasks and measures of attention. In addition, both the uncinate fasciculus and anterior limb of the internal capsule (which contains the same axons as the anterior corona radiata) play key roles in emotional behaviors.

The uncinate fasciculus, is a critical part of the ventro-lateral limbic network which links the amygdala, a limbic structure, with the orbitofrontal cortex via the anterior temporal pole. The amygdala, in turn, has connections with the dorsomedial thalamus, which projects back to the prefrontal cortex (including orbitofrontal cortex) as well as to the hypothalamus (the principle nuclear complex regulating autonomic function) and to the output nuclei of the ANS in the brainstem (dorsal motor nucleus, nucleus of the solitary tract and nucleus ambiguus).

The other frequently injured structure, the anterior limb of the internal capsule (IC-AL) and anterior corona radiata links the prefrontal cortex, including orbitofrontal cortex, to mesolimbic structures such as the nucleus accumbens, hypothalamus and thalamus, including both the dorsomedial thalamus and the midline “limbic” thalamic nuclei. The IC-AL is the principle means by which prefrontal cortex and thalamic nuclei such as the central median/parafascicular nuclear complex and the dorsomedial nuclei interact (Nadeau and Crosson, 1997).

## EFFECT OF mTBI ON BEHAVIOR

While cognitive and emotional symptoms of mTBI are often transient, mTBI has been associated with several persistent behavioral changes including changes in mood and emotional reactivity, the development of attention deficit hyperactivity disorder and conduct disorders, and decrements in cognitive performance such as working memory. Many of these behavioral disorders are associated with alterations in frontal – subcortical networks. In the United States military, discharge rates for individuals with mild, moderate and severe TBI were higher than those discharged without history of TBI during the year 1992 (Ommaya et al., 1996). Elevated rates of drug and alcohol use (2.6 times), and criminal discharge (2.7 times) status were found for the mTBI group compared with the general population. Further, both civilian and military populations evidence similar psychiatric profiles (though with differing incidence) subsequent to mTBI, including elevated reports of PTSD and depression. In a military population, Hoge et al. (2008) reported 43.9% of service members experiencing loss of consciousness were screened positive for PTSD and 24.8% screened positive for depression. Similarly, Lew et al. (2007), reported 42% of Operation Enduring Freedom veterans with a history of mTBI also had PTSD symptoms. Further, the same study reports the following percentages of symptom complaints subsequent to mTBI: 74% anxiety, 66% depression, 76% mood swings, and 84% irritability.

The consequences of these mood/emotion changes appear to be substantial. According to a study of 100 patients with mild to severe TBI who were followed up to 5 years post-injury, outcomes in vocation, personal relationships and independence are predicted by ongoing depression as well as anxiety. These relationships are, notably, independent of pre-morbid psychiatric status (Whelan-Goodinson et al., 2008). Understanding how depression, anxiety, and other emotional processing changes occur with mTBI is important in determining possible interventions.

Anatomic alterations of the brain have been linked to alterations in mood in various brain injury populations. For example, a study investigated 42 political detainees and contrasted 16 with TBI (blunt-force) with 26 without TBI. Those with TBI had thinner prefrontal-temporal cortices (e.g., assessed by surface reconstruction and measurements of cortical thickness using cortical surface-based analysis) with specific changes in left dorsolateral prefrontal and bilateral superior temporal cortex. Further, these changes were related to depression scores (Mollica et al., 2009). In a sample of blast-related mTBI patients, Matthews et al. (2011), compared patients with and without major depression (11 MDD and 11 non-MDD patients). They found that the MDD group had significantly lower DTI-FA values in the left and right anterior limb of the internal capsule. This study also had a functional component. In response to an emotional face-matching task that included angry, fearful, and happy faces, those TBI patients with MDD showed greater bilateral amygdala activation (using fMRI) than did the TBI patients without MDD, possibly suggesting loss of inhibitory input from phylogenetically newer areas to this ventrolateral-limbic system. In contrast, Levin et al. (2010), studying 37 veterans with TBI and 15 without TBI found no relationship between mood measures, cognitive performance, and DTI-FA values. The contrasting results may reflect methodological differences. Specifically, Matthews et al. (2011), used a reactivity model with affective stimuli (emotional faces presented and regional activation assessed with fMRI), whereas Levin et al. (2010) used self-report mood scales. Also, sample selection was different between the two studies, with Matthews et al. (2011), measuring patients already diagnosed with major depressive disorder versus those without major depressive disorder.

Although mTBI has impacts on the functional behavior of the brain, evaluating this impact has traditionally been challenging. For example, although Gaetz and Bernstein (2001) concluded that standard clinical EEG is not useful for the assessment of mTBI, they thought that combining EEGs, ERPs, and cognitive testing might enable accurate assessment. Behavioral outcome may be relevant to EEG profile. Meaning, if mTBI affects systems that modify emotional responding, in patients that manifest mood symptoms, different physiological profiles than patients who do not manifest mood symptoms may be detected. Rabe et al. (2006) compared patients with PTSD and without after a motor vehicle accident, and, using spectral analyses of EEG, found significant differences in alpha bandwidth power over the right parieto-temporal cortex. In a potentially contradictory EEG evaluation of patients with PTSD and healthy controls, Kim et al. (2012), using a measure of non-linear interdependence on resting EEG, found that patients with PTSD exhibited an increase in non-linearity in the left hemisphere. The combination of structural and functional evaluations in mTBI and PTSD has been scarcely reported in the literature.

## EMOTION

As previously stated, reports of affective and emotional consequences of mTBI are wide spread (Ommaya et al., 1996; Lew et al., 2007; Whelan-Goodinson et al., 2008; Mollica et al., 2009; Levin et al., 2010). Independent of TBI research, a common thread present throughout theoretical conceptualizations of emotion has been the construct of arousal (central and peripheral). At the turn of the 20th century, William James and Carl Lange postulated that physiological feedback from the body is important in emotional experience. Based on Claude Bernard’s classic research, Darwin (1872) stated, “… when the heart is affected it reacts on the brain; and the state of the brain again reacts through the pnuemo-gastric [vagus] nerve on the heart; so that under any excitement there will be much mutual action and reaction between these, the most important organs of the body.” Many of the body’s physiological functions are controlled by the ANS and to fully understand emotional processing, we must understand the central neural circuits that regulate the ANS. The limbic system, and its connections to structures such as the hypothalamus, plays an important role in the control of the ANS; interruptions in the interconnectivity of these systems may impact emotional experience.

Affect, cognition (executive functions), and ANS functions depend upon shared white matter pathways that are often injured by trauma. There appears to be a high likelihood that damage to specific white matter damage in patients with mTBI may explain some of the variance in the presence of emotional disorders. Further, while limited data exist correlating mood measures in this population to specific white matter regions, these relationships have not been studied systematically. However, autonomic functions are an important component of emotional behaviors. This relationship may be especially relevant in patients with mTBI, since due to the increased incidence of PTSD and the expression of symptoms associated with alterations of ANS functions.

Emotional experiences are not unique to humans. Animals display behaviors consistent with a fear-response when confronted with a threatening situation just as do humans. From an evolutionary perspective, emotions reflect, to some extent, the functions of phylogenetically older systems. Telencephalic structures like the amygdala (Davidson et al., 1999), cerebral cortex (Heilman and Gilmore, 1998), and basal ganglia (Gray et al., 1997) have been the focus of studies of human emotion. However, phylogenetically older structures such as the thalamus, hypothalamus, and reticular formation play an important role in cognition, attention, and arousal and these more primitive systems have an important role in emotional experience and behaviors (Tucker et al., 2000).

Jackson (1879) introduced the principle of hierarchic integration through inhibitory control (dissolution). Specifically, this precept refers to the modulation, inhibition, and elaboration of older brain systems, rather than the replacement of said mechanisms. Many older brain systems continue to play an important role in higher order cognitive and emotional functions. Thus, there are reciprocal excitatory and inhibitory influences of the cerebral cortex with more primitive systems and intact communication amongst these systems is necessary for the production of normative emotional experiences and behaviors. In the context of mTBI, it is possible that these hierarchical relationships may be disrupted by damage to key white matter structures resulting in abnormal emotional behaviors, such as PTSD.

## CEREBRAL INTERACTIONS IN AUTONOMIC NERVOUS SYSTEM CONTROL

Early research on the ANS recognized the importance of the cerebrum in modulating response to stressors, and prefrontal networks in particular have been strongly implicated in cerebral control of the ANS (Loewy, 1991). In comparative neuroscience research, consistent results have demonstrated that the prefrontal cortex can inhibit activation of the sympathetic nervous system, possibly by modulating parasympathetic activity (Hardy and Holmes, 1988; Owens and Verberne, 2001) including baroreceptor reflexes (Resstel et al., 2004). In a review of the literature on cortical control of the cardiovascular system, Verberne and Owens (1998) cite evidence from anatomical, lesion, and electrical stimulation studies that the medial prefrontal cortex is preferentially involved in modulating sympatho-inhibitory responses. They concluded that medial prefrontal cortex has consistently been demonstrated to be a depressor area with respect to ANS function (e.g., activity is associated with decreased blood pressure). While functionally this relationship exists, the full extent of anatomical bridges between aspects of prefrontal cortex and nuclei involved in ANS control is not fully known. Anatomically, there are cortical projections to the nucleus of the solitary tract, which receives cardiopulmonary afferents by way of the vagus nerve. These interconnections are thought to be involved in blood pressure, vasomotor, and heart rate regulation through baroreceptor reflexes (a control system that modulates and coordinates cardiovascular responses to changes in blood pressure). In humans, cardiovascular arousal (increased heart rate and mean arterial pressure) has been associated with decreased rCBF (PET) in prefrontal cortex (Critchley et al., 2000).

Both animal and human studies also suggest that there is lateralized differentiation in the control of the ANS. For example, electrical stimulation of right and left insular cortex has been demonstrated to yield tachycardia and bradycardia, respectively (Oppenheimer et al., 1992; Oppenheimer et al., 1996). Similarly, studies using hemispheric anesthesia (Wada testing) have revealed that heart rate variability attributes are modified by right and left hemisphere inactivation in a manner which suggests left hemispheric lateralization for parasympathetic control (Yoon et al., 1994). However, right hemispheric infarction in the rat is associated with elevated catecholamines, with corresponding increases in heart rate and blood pressure (Hachinski et al., 1992). Right hemisphere stroke in humans is more frequently associated with supra ventricular tachycardia than in left hemisphere strokes (Lane et al., 1992). This paradoxical relationship may be related to sympathetic disinhibition and the volume of right insular cortex infarction correlates well with the degree of catecholamine elevation (Sander and Klingelhöfer, 1995). Right insular lesion is also associated with greater probability of death within one year, possibly related to pathological sympathetic activation of the cardiovascular system (Colivicchi et al., 2005). Also, right brainstem infarct is more likely to result in death (Bogousslavsky et al., 1990). Furthermore, the cortical and subcortical brain systems involved in the regulation of autonomic activity are also involved in the regulation of behavioral processes including executive functions (Gianaros et al., 2004), affect (Damasio et al., 1990; Berntson et al., 1998), and state regulation (Porges, 1995, 2007).

## POST TRAUMATIC STRESS DISORDER

Major depressive disorder and PTSD are two of the more common mental illnesses diagnosed after mTBI. Could specific white matter damage lead to alternations in ANS and increase vulnerability to disorders such as PTSD.

To model the potential impact of mTBI on PTSD, it is useful to consider the pathophysiology of PTSD and the functional systems that are involved in PTSD. PTSD symptoms can be grouped into three categories: (1) re-experiencing symptoms (e.g., flashbacks, bad dreams and frightening thoughts), (2) avoidance symptoms (e.g., feeling emotionally numb, guilt, depression, worry, anhedonia), and (3) hyperarousal (e.g., hyper-reactive, easily startled, anger outbursts, difficulty sleeping). Functionally, re-experiencing may be a form of emotional memory perseveration and avoidance may be an aspect of an adaptive defense system reflected in immobilization and derivative of an ancient phylogenetic circuit.

Atypical emotional processing has been reported in PTSD. For example, in an fMRI study of men and women patients with PTSD had greater bilateral amygdala activation to photographs of fearful facial expressions than healthy controls (Felmingham et al., 2010). PTSD is also associated with decreased heart-rate variability, suggesting parasympathetic withdrawal (Tan et al., 2011). McTeague et al. (2010) investigated reactivity to loud acoustic stimuli while imagining threatening and neutral events in individuals with and without trauma exposure. Trauma exposure was related higher physiological arousal reflected in an enhanced startle reflex. A further investigation of the data, evaluating participants with severe trauma or multiple traumas, identified a physiological hyporeactivity. The study authors hypothesized that this may reflect sustained high stress and egregious persistent negative affectivity, compromising defensive responses. However, the hypoactivity may be related to injury to the brainstem mechanisms that mediate this reflex or to the hemispheric networks that normally enhance the startle reflex and the connections within the networks. It may also reflect differences of involved functional cerebral systems, for example the laterality impacts that have heretofore not been addressed. Even in the absence of physical trauma to the brain, psychological trauma may change how the brain works (e.g., McTeague et al., 2010).

In a study of rats that underwent psychological stress, glutamate levels were decreased in the medial prefrontal cortex when compared to controls. The depressed glutamate levels suggest decreased excitatory tone without loss of neuronal integrity in this region (Knox et al., 2010). Activity in the medial prefrontal cortex is associated with suppression of the sympathetic nervous system (Verberne and Owens, 1998). There is only one published study of white matter integrity shifts in patients with PTSD, but without TBI (Schuff et al., 2011, at the VA Medical Center in San Francisco). In this study of 17 male veterans with PTSD and 15 healthy controls, the investigators measured DTI and regional blood flow (using arterial spin labeling) and found that patients with PTSD had fractional anisotropic reduction in white matter regions including areas within the prefrontal cortex, posterior internal capsule, and insula (an important component of the central control of autonomic resources) as well as increased activity in the right temporal and parietal areas. The latter has been shown to be associated with increased sympathetic arousal and negative emotions in other populations. The loss of axonal integrity with psychological stress is surprising and, it remains to be seen if this is a reproducible finding and what might be inducing these changes. For example, these findings may be due to increased cortisol levels.

In a preliminary treatment study for PTSD conducted at Walter Reed Army Medical Center, Roy et al. (2010) noted that their population had relatively severe PTSD symptoms and was treatment resistant. The authors suggested this may be the result of mTBI and planned to answer this question by comparing subjects with blast injury exposure and no PTSD, subjects with PTSD but no blast exposure, subjects with blast exposure and PTSD, as well as normal healthy controls. Using fMRI, these investigators presented compelling data demonstrating that simulated exposure to combat environments elicits changes in the amygdala. However, the sample size for this study limits inference and the ability to address more specific questions (e.g., differences between various subgroups within PTSD).

## SYNTHESIS: PTSD, TBI, AND AUTONOMIC BEHAVIOR

Mechanistically, PTSD can be conceptualized as a deficit in autonomic adaptation, an incongruity in physiological state and environmental demands. High vigilance and preparedness for fight or flight are potentially adaptive in the appropriate environment, for example, in the context of a military theater. However, while an individual is sitting in an office, shopping in a supermarket, preparing for sleep at home, or participating in other activities within the confines of safety, a fight or flight state is maladaptive. Hyper-reactivity would be counter to the demands of the situation, resulting in significant psychosocial distress and often deteriorating relationships. Autonomic dispositions can be influenced by disruption in the hierarchically organized systems of the central autonomic network. This could be due to physical insult (e.g., mTBI). The ventrolimbic (orbitofrontal cortex, uncinate fasciculus, anterior temporal lobe, amygdala, etc.) and thalamo-cortical pathways are critical in the processing of normal and abnormal emotional behaviors (Mayberg, 1997; Blumberg et al., 2002; Phillips et al., 2003). Some of these areas are vulnerable to damage in mTBI (Niogi et al., 2008). The presence of PTSD may also reflect pre-injury personality (e.g., hostility, see Williamson and Harrison, 2003), premorbid behavior of relevant systems (e.g., genetic predisposition for both PTSD and heart rate variability, see Kupper et al., 2004; Koenen, 2007) or a combination of factors. Thus, people in the context of mTBI and PTSD are often exposed to situations that would be likely to influence the development of PTSD, independent of mTBI.

To help inform our understanding of ANS function and the consequences of compromising cortical and specifically frontal regulation of subcortical centers of autonomic control and this relationship to PTSD, we turn to a hierarchical model of ANS organization and function, the Polyvagal Theory. According to the Polyvagal Theory (Porges, 1995, 2007, 2009; Porges and Furman, 2011), which relates ANS function to behavior (e.g., social engagement, emotional regulation), neurophysiological mechanisms and phylogenetic shifts in neural regulation of the ANS are at the core of emotional experience. The ANS, including both target organ afferent and efferent pathways, is important to our understanding of behavior. The phylogeny of the vertebrate ANS identifies in mammals a unique reciprocal influence between the heart and face via the central nervous system pathways. Consistent with historical observations linking of physiological response to emotion and behavior (e.g., Darwin), integrative theories of central nervous system and ANS function have recently emerged in the neuroscience literature. As understanding of these neurophysiological mechanisms has improved, we have been able to predict more precisely the interaction of ANS state and behavioral response. The Polyvagal Theory stipulates that primary emotions are related to ANS functions and this relationship is dependent on communication across multiple structures, hierarchically linked. Sequencing of this system is informed by the Jacksonian construct of dissolution. Dissolution is the functional notion of evolution in reverse, in which phylogenetically newer cortical structures are inhibitory over older subcortical structures and that the system works, in part, through releasing inhibition (or, hierarchical integration). Because of the dependence on communication between these systems on fast regulation (e.g., dampening autonomic reactivity), the white matter damage associated with mTBI may be particularly problematic.

Our emotional/engagement systems are dynamic and operate in a continuous manner and these systems can be thought of in terms of balance states across interconnected functional modules. It is helpful to discuss these modules as discrete systems, but they are not. Rather, similar structures and functional entities interact to produce behavior depending on input from the environment and internal factors, including arousal, personality, past experience, neural damage, etc. In the context of autonomic adaptation and social engagement, we must be able to achieve eye contact to properly engage someone, read and produce facial expressions, and activate the expression and reception of prosodic speech. These behaviors are supported by visceral homeostasis. This visceral homeostasis may be modified by the detection of threats. As a threat becomes salient, according to the Polyvagal Theory, as mammals we have at our disposal two systems of response, fight/flight strategies (mobilization) and death feigning/shutdown strategies (immobilization). These states can be adaptive or maladaptive depending on context.

The determination of safety is a critical function of the nervous system. The perception of safety drives whether or not people are amenable to social engagement or likely to engage in a defensive disposition. However, the determination of risk may not be at the conscious level (see discussion of neuroception below). Perhaps the core deficit in PTSD is a defensive reactive strategy to features in the environment outside of awareness. This defensiveness is often coupled to an inability to shift to a safe disposition. Similar social actions, which to others provide salient signals of “safety,” may be perceived as threat for the individual with PTSD. The context for an individual is interpreted through memory and emotional cues. In order to effectively shift from defensive dispositions to socially engaging dispositions, the individual needs to determine safety and then inhibit the more primitive limbic structures that control flight, flight, or freeze behaviors.

The determination of environmental threat is a multi-system process. Porges (2003) argues that the evaluation of risk in the environment is not within the volitional control of an animal, and is a series of permutations that is automatic, a construct he describes as neuroception. However, experience/learning impacts this process of threat detection. Further, volitional strategies can impact this process. Contextual features in the environment interact with endogenous variables to influence the detection of threat and the stage of physiological interaction. If the features in an environment are evaluated via neuroception as safe, prosocial behaviors may occur. If an environment is evaluated as dangerous, one of two defensive strategies will occur. The detection of safety is a critical variable for positive social experiences and an inability to detect features in the environment as safe underlies many psychiatric conditions. Thus, the human must perform two activities for successful social behaviors: (1) assess threat, and (2) if the environment is evaluated via neuroception as free of threat, inhibit the more primitive limbic structures that control fight, flight and freeze behaviors. Threat detection is supported by networks in sensory, association cortices as well as polymodal areas including areas of the temporal cortex, fusiform gyrus (face detection in the right hemisphere), and their interconnections to amygdala (Adolphs, 2002; Pessoa et al., 2002; Winston et al., 2002).

In mTBI, PTSD may reflect altered defensive responding of the response systems to environment challenge, a chronic shift in ANS state/disposition. Autonomic dispositions may be described in terms of phylogenetically ordered environmental responses. Based on the Polyvagal Theory (Porges, 1995, 2007), there are three hierarchically ordered response systems to environmental challenge. The nervous system attempts to match neurophysiological resources to support each adaptive biobehavioral response system to support the neuroceptive threat level. Note that the symptoms of TBI/PTSD are primarily expressed as a deficit in social engagement, which when functioning, regulates and dampens circuits involved in defense that are, when disinhibited, expressed as either fight/flight or immobilization (e.g., behavioral shutdown) or a combination of both.

We can conceptualize this hierarchically as follows: *Stage 1, Social Engagement* – the most phylogenetically advanced adaptation system underlies our social communication behaviors (modulating facial expression, vocalization, and listening). This system is dependent on the myelinated vagus and serves to foster calm behavioral states by inhibition of sympathetic influences to autonomic control by dampening the HPA axis (Bueno et al., 1989). Modulation of visceral state via activation of the brainstem nucleus ambiguous, which has direct vagal input, promotes social engagement. With the detection of threat, mobilization or immobilization behaviors are recruited. *Stage 2, Fight or Flight –* the mobilization system is dependent on the function of the sympathetic nervous system, increased blood pressure, decreased heart-rate variability enables proper execution of this activity. *Stage 3, Immobilization –* immobilization is the most phylogenetically primitive component of our defensive systems. It is dependent on umyelinated or “vegetative” vagus. Most vertebrate organisms have this system.

Traumatic brain injuries could influence the ability of someone to inhibit limbic structures and this failure may be induced by a loss of connections with phylogenetically newer areas of cortex (i.e., prefrontal cortex). A person with PTSD may be able to consciously express that a given situation is safe but be unable to shift viscerally to an appropriate autonomic state. Likewise, they may be unable to properly assess safety in their environment when in a hypervigilant autonomic state. The physiological reaction to risk perception is metabolically costly. McEwen’s concept of allostatic load applies for the patient with PTSD (McEwen and Wingfield, 2003). Although the defensive response is adaptive in short-term threat situations, it is a chronic stress over long periods. The chronic defensive mobilization observed in patients with PTSD leads to deterioration of health and social relationships. Treatments for PTSD, and other anxiety disorders, tend to focus on reducing neuroceptive determination of threat. For example, flooding, a behavioral theory concept, characterized by intense exposure to erroneously threatening stimuli, is effective because there are no deleterious consequences associated with that exposure. The treated individual learns to decouple the threat response from the problem stimuli. We see from treatment methods, such as Cognitive Processing Therapy, which teach awareness of emotional states, that there is also an attempt to engage volitional systems to challenge automatic thoughts. Though non-neurobiologically driven theories, the success of this kind of therapeutic approach suggests that neuroception may be modifiable by intentional systems, possibly mediated by the prefrontal cortex.

The autonomic disruption in patients with PTSD is a critical health issue. Autonomic behavior (e.g., respiratory sinus arrhythmia) is an independent predictor of mortality (Tsuji et al., 1994) and, specifically, heart disease (Kubzansky et al., 2007). It is not entirely clear what factors contribute to the development of heart disease in this population. But, one possible explanation is effects from chronic parasympathetic nervous system withdrawal (Porges, 2009). In a recently published twins study on combat trauma exposure and PTSD, Shah et al. (2013) studied 20 twin pairs that were discordant for PTSD. They demonstrated reduced heart rate variability for the twins diagnosed with PTSD. Patients with remitted PTSD were not different from their twin on these measures. In this study, combat exposure was inversely associated with heart rate variability, a relationship that was mostly eliminated when adjusting for current PTSD. Thus, the negative health impacts of combat exposure on autonomic state may possibly be largely reversed with successful treatment of PTSD. However, PTSD is also associated with systemic inflammation, perhaps due to increased allostatic load. Gola et al. (2013) showed increased plasma cytokine levels and spontaneous and lipopolysaccharide stimulated cytokine production, suggesting low-grade systemic inflammation in PTSD. As of yet, inflammatory reversals have not been demonstrated with successful PTSD treatment and this is another poor physical health predictor.

Recently, efforts to treat PTSD by modulating sympathetic nervous system activity have shown promising results. Chronic increases of catecholamines, including epinephrine and norepinephrine, support increased sympathetic load and this has been demonstrated in patients with PTSD (Lemieux and Coe, 1995). Norepinephrine focuses attention and enhances memory (e.g., ADHD pharmacotherapy interventions such as atomoxetine increase norepinephrine levels). Norepinephrine levels in cerebrospinal fluid have been correlated with the severity of PTSD symptoms in patients with PTSD (Geracioti et al., 2001). Lipov et al. (2008) demonstrated and later reviewed (Lipov and Kelzenberg, 2012) successful use of stellate ganglia block to treat PTSD symptoms. A stellate ganglia block prevents lateralized sympathetic nervous system input from the stellate ganglia to the periphery and there is some evidence that is may also affect intracranial activity.

Acutely, it is possible that stimulants and other adrenergic up-regulation increase the likelihood of developing PTSD. In the military, stimulants are regularly used to improve attention and performance (e.g., Gore et al., 2010). Research on the effect of stimulant use during stressor exposure may be useful to understand medication/environment variables affecting healthy outcomes in combat situations. Subsequent to trauma, during exposure therapy treatment, the use of propranolol, a beta-blocker that reduces blood pressure and also passes the blood-brain barrier, has been used as an effective treatment adjuvant. Propanolol, which reduces norepinephrine levels, reduced psychophysiological responses and severity of PTSD symptoms (Poundja et al., 2012).

Efforts to prevent the development of PTSD, to treat it after its development, and to understand the contributions of mTBI to increased vulnerability hinge on understanding autonomic and emotional interactions in shifting between adaptive and maladaptive physiological states. Baseline assessments of personality, which are already done in the military, with addition of autonomic reactivity evaluations to emotional stimuli may prove useful in the prediction of PTSD vulnerability. Depending on where soldiers fit in a Yerkes-Dodson like continua, combat effectiveness could be enhanced and the probability of PTSD development mitigated by pre-combat propranolol administration.

In summary, psychiatric disorders such as PTSD have been associated with changes in regional activation patterns and white matter integrity. The presence of these changes makes it difficult to distinguish between alterations in brain structure and function related to the psychological impact of exposure to a traumatic experience – i.e., the usual etiology of PTSD – and those related to physical trauma, or if there is an interaction effect. It may be that white matter changes attributed to mTBI in previous studies reflect, in part, an interaction effect that leads to psychiatric illness. For example, after an individual experiences a mTBI and subsequently develops PTSD, the latter may cause changes in white matter integrity. In order to distinguish between these conditions, future research will need to integrate methodologies that allow us to identify damaged systems through a variety of neuroscience technologies (e.g., histopathology, neuroimaging, neurochemistry) and relate these metrics to abnormal behavioral and physiological expression such the disruption of autonomic regulation in response to emotional stimuli.

## Conflict of Interest Statement

The authors declare that the research was conducted in the absence of any commercial or financial relationships that could be construed as a potential conflict of interest.
